# Organizing Pneumonia in a Patient with Quiescent Crohn's Disease

**DOI:** 10.1155/2016/8129864

**Published:** 2016-06-20

**Authors:** Satoshi Tanida, Masaya Takemura, Tsutomu Mizoshita, Keiji Ozeki, Takahito Katano, Takaya Shimura, Yoshinori Mori, Eiji Kubota, Hiromi Kataoka, Takeshi Kamiya, Takashi Joh

**Affiliations:** ^1^Department of Gastroenterology and Metabolism, Nagoya City University Graduate School of Medical Sciences, Nagoya, Aichi Prefecture 467-8601, Japan; ^2^Department of Respiratory Medicine, Allergy and Clinical Immunology, Nagoya City University Graduate School of Medical Sciences, Nagoya, Aichi Prefecture 467-8601, Japan

## Abstract

A 64-year-old man with Crohn's disease (CD) was admitted to our hospital due to moderate risk of pneumonia while receiving scheduled adalimumab maintenance therapy. Symptoms remained virtually unchanged following administration of antibiotics. A final diagnosis of organizing pneumonia (OP) was made based on findings of intra-alveolar buds of granulation tissue and fibrous thickening of the alveolar walls on pathological examination and patchy consolidations and ground glass opacities on computed tomography. Immediate administration of prednisolone provided rapid, sustained improvement. Although a rare complication, OP is a pulmonary manifestation that requires attention in CD patients.

## 1. Introduction

Inflammatory bowel diseases (IBDs), such as ulcerative colitis (UC) and Crohn's disease (CD), often affect the entire digestive system and organs, but extraintestinal manifestations account for approximately 21–41% of IBD cases [1, 2]. Noninfectious pulmonary diseases associated with CD are uncommon. These associations are generally considered to arise from extraintestinal manifestations of the disease itself and adverse effects to therapeutic drugs prescribed to ameliorate inflammation [3].

Organizing pneumonia (OP) is a particular form of pneumonia presenting as diffuse interstitial lung disease that affects the distal bronchioles, respiratory bronchioles, alveolar ducts, and alveolar walls [4–6]. OP appearing with CD is extremely rare. Only a single report has described nonresolving pneumonia in 3 CD patients receiving immunosuppressive agents: 2 patients had pneumonia presenting as noncaseating granuloma with accompanying giant cells and 1 patient had OP [7]. Here, we report a rare case of OP in a CD patient who had been in remission after receiving 5-aminosalicylic acid and adalimumab (ADA).

## 2. Case Report

This patient was a 64-year-old man who had previously undergone partial ileal resection due to intestinal obstruction and partial peritonitis at the age of 30. He had since been admitted to other hospitals because of repeated temporary obstructions at the anastomotic site. At the age of 62, the patient was referred to our hospital with recurrent severe abdominal pain and exacerbation of CD. The patient had no history of lung disease, occupational exposures, or extraintestinal manifestations and no family history of CD. Physical examination revealed the following: body temperature, 39.2°C; hemoglobin oxygen saturation as measured by pulse oximetry, 99%; and blood pressure, 100/66 mmHg.

**Figure 1:**
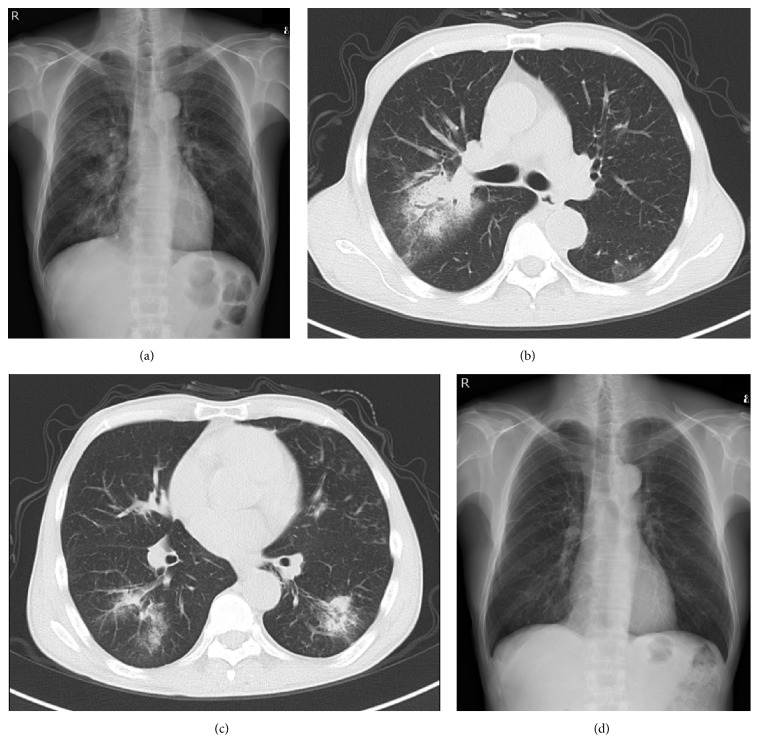
(a) Chest radiograph shows patchy bilateral consolidation in the middle lobe and bilateral lower lobes. (b, c) Chest computed tomography reveals patchy consolidation, ground glass opacities, and small nodular opacities in the middle (b) and bilateral lower lobes (c). (d) Follow-up chest radiograph after 3 weeks shows resolution of abnormal findings.

**Figure 2:**
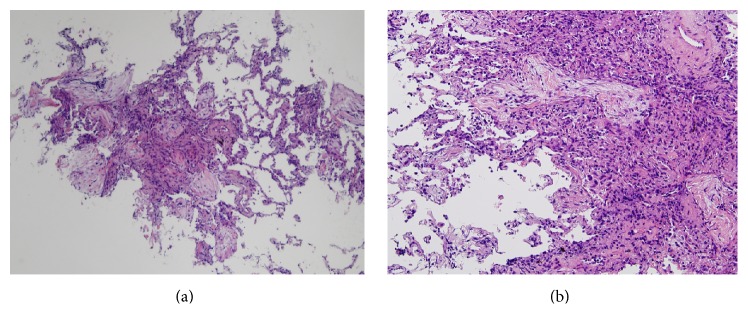
Pathologic examinations of transbronchial lung biopsy specimen with hematoxylin and eosin stain ((a) ×40 and (b) ×100) show intra-alveolar buds of granulation tissue and fibrous thickening of the alveolar wall.

**Figure 3:**
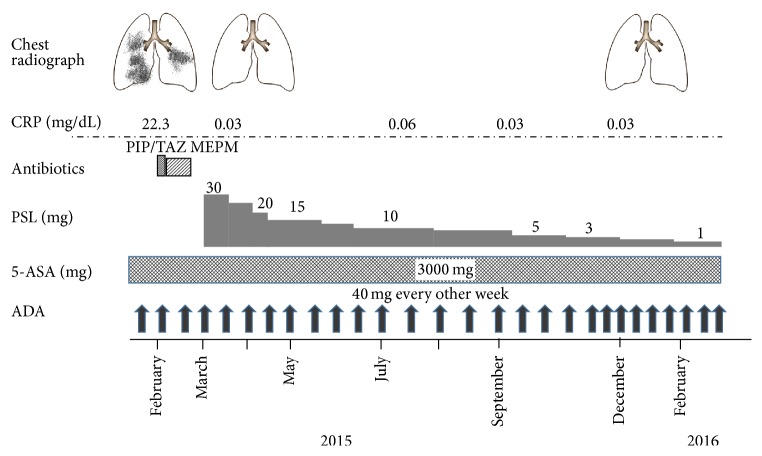
The clinical course of the patient, a 64-year-old man with Crohn's disease. Progressive pulmonary symptoms and manifestations were seen during scheduled ADA maintenance therapy. Treatment with prednisolone induced rapid, sustained improvements. CRP: C-reactive protein; PIP/TAZ: piperacillin/tazobactam; MEPM: meropenem; PSL: prednisolone; 5-ASA: 5-aminosalicylic acid; ADA: adalimumab.

A small bowel series showed longitudinal ulcers and strictures at the ileal anastomotic site. Colonoscopy and pathological examination of a biopsied ulcer specimen showed moderate to severe inflammatory cell infiltrates with some epithelioid granulomas in the lamina muscularis mucosae and submucosa. Based on these findings, CD was initially diagnosed. ADA induction therapy was then initiated at 160/80/40 mg every other week [8] and afforded complete remission. Two years later, the patient was admitted to our hospital due to rapid exacerbation of productive cough, accompanied by fever of up to 39.2°C, and findings on chest radiography of patchy bilateral consolidation and ground glass opacities (Figure 1(a)). Laboratory investigations showed the following: white blood cell count, 13,800/*μ*L; red blood cell count, 386 × 10^4^/*μ*L; hemoglobin, 10.7 g/dL; total protein, 5.3 g/dL; albumin, 2.1 mg/dL; aspartate aminotransferase, 58 IU/L; alanine aminotransferase, 34 IU/L; C-reactive protein, 22.2 mg/dL. Low albuminemia was due to severe inflammation and loss of appetite. Negative results were obtained from tests for influenza virus types A and B,* Aspergillus* antigen, (1→3)-*β*-D-glucan, QuantiFERON, and cytomegalovirus antigenemia C-7HRP. Sputum cultures isolated* Streptococcus pneumoniae* and *α*-hemolytic streptococcal species (Table 1). Chest computed tomography (CT) revealed patchy consolidations, ground glass opacities, and small nodular opacities, predominantly in the middle, lingula, and bilateral lower lobes (Figures 1(b) and 1(c)). Based on these findings, we considered a diagnosis of bacterial pneumonia due to streptococcal species. Despite antibiotic treatment with intravenous piperacillin-tazobactam and meropenem and supportive care for 14 days, respiratory symptoms remained unimproved and radiographic findings gradually deteriorated. A transbronchial lung biopsy from a right lung lesion showed fibrous thickening of the alveolar wall and intra-alveolar buds of granulation tissue associated with fibroblasts (Figure 2). Based on these findings, a final diagnosis of OP associated with CD was made though postinfectious OP could not be completely ruled out. Upon starting prednisolone at a dose of 30 mg/day, dramatic clinical improvement was observed and complete radiographic clearing of the lung infiltrates was confirmed within 3 weeks (Figure 1(d)). The prednisolone dose was gradually tapered to 1 mg/day (Figure 3). The patient has remained well for a year.

## 3. Discussion

CD often shows extraintestinal manifestations such as uveitis and ankylosing spondylitis [9]. On the other hand, primary lung involvement is rare and reportedly includes bronchiolitis obliterans organizing pneumonia (BOOP), pulmonary interstitial emphysema, desquamative interstitial pneumonia, nonspecific interstitial pneumonia, fibrosing alveolitis, and eosinophilic pneumonitis [2, 3]. Lung involvement can also be secondary to the drug used to treat CD [10]. OP is the most commonly reported parenchymal manifestation of IBD, particularly UC [11]. In this type of idiopathic diffuse interstitial lung disease, granulation tissue obstructs the alveolar ducts and alveolar spaces and chronic inflammation arise in the adjacent alveoli [5, 6]. Pulmonary interstitial emphysema is characterized by air trapping outside the normal air passages and inside the connective tissue of the peribronchovascular sheaths, interlobular septa, and visceral pleura; this disease entity is more frequent in premature infants who require mechanical ventilation for severe lung disease [12]. Desquamative interstitial pneumonia is a chronic lung inflammation characterized by mononuclear cell infiltration of the airspaces. It occurs almost exclusively in current or former cigarette smokers [13]. Fibrosing alveolitis is characterized by inflammation and thickening of the alveolar walls and usually occurs in individuals over the age of 40 years [14]. Eosinophilic pneumonitis is a disease characterized by accumulation of eosinophils in bronchoalveolar lavage and lung tissue [15]. In the present case, the final diagnosis of OP associated with CD was made based on the pathological and CT findings and the clinical course [16–18]. However, in terms of the clinical features, postpneumonic OP and secondary OP, these are actually fairly similar. Differentiating postpneumonic OP from secondary OP based on the radiographic findings was difficult. We initially diagnosed acute bacterial pneumonia due to streptococcal species based on the rapid development after disease onset and the positive result of sputum cultures for* Streptococcus pneumoniae* and *α*-hemolytic streptococcal species. Despite the use of wide-spectrum antibiotics, respiratory symptoms persisted and findings on chest radiography gradually deteriorated. In general, in the early period of postpneumonic OP, antibiotics therapies are partially effective and yield some degree of improvement in symptoms and radiological findings [19, 20]. Based on the above, we concluded that secondary OP was more likely than postpneumonic OP.

Lung disease is known to sporadically occur after long-term use of 5-aminosalicylic acid [21] and anti-tumor necrosis factor (TNF)-*α* antibodies [22, 23]. Consensus is currently lacking regarding a definitive approach to diagnosing drug-induced lung disease. In a few cases, drug-induced lung disease can show a clinical course and radiographic findings like OP [24, 25]. In the present case, at disease onset, the patient was immediately given prednisolone, which provided rapid and sustained improvement. The prednisolone was subsequently tapered while ADA and mesalazine were continued. As a result, the patient has remained well without recurrence of OP. These results suggest that this lung disorder was unlikely to present a drug-induced lung disease and was instead more likely to be OP associated with CD.

For patients with progressive symptoms of cryptogenic organizing pneumonia (COP) and diffuse radiographic changes, initial therapy with oral glucocorticoids is recommended for promising clinical outcomes [26, 27]. However, one case has been described in which initial treatment with glucocorticoids led to poor outcome, whereas a dramatic response to infliximab was seen. In the present case with OP occurring with CD in clinical remission, we selected prednisolone therapy because we considered that TNF-*α* was unlikely to have played a pivotal role in the development of OP and because the patient had received ADA treatment. Interestingly, OP cases have not been known to be associated with the disease activity of IBD [2, 11, 28].

In conclusion, although OP is a rare complication, due vigilance is required regarding the occurrence of pulmonary symptoms and manifestations in CD and IBD patients.

## Figures and Tables

**Table 1:** Laboratory findings on admission.

		Normal range
*Hematology*		
WBC	13600/*μ*L	3,600–9,600/*μ*L
RBC	386 × 10^4^/*μ*L	400–552 × 10^4^/*μ*L
Hb	10.7 g/dL	13.2–17.2 g/dL
PLT	193 × 10^3^/*μ*L	148–339 × 10^3^/*μ*L
*Serum biochemistry*		
TP	5.3 g/dL	6.7–8.3 g/dL
Alb	2.1 g/dL	4.0–5.0 g/dL
AST	58 U/L	13–33 U/L
ALT	34 U/L	6–30 U/L
LDH	303 U/L	214–466 U/L
BUN	19.2 mg/dL	8–22 mg/dL
Cre	0.9 mg/dL	0.6–1.1 mg/dL
Na	138 mmol/L	138–146 mmol/L
K	4.4 mmol/L	3.6–4.9 mmol/L
Cl	99 mmol/L	99–109 mmol/L
CRP	22.2 mg/dL	≤0.30 mg/dL
*Serologic tests*		
Influenza virus types A and B	Negative	
Antigenemia (C-7HRP)	Negative	
Aspergillus antigen	Negative	
(1→3)-*β*-D-glucan	Negative	
QuantiFERON	Negative	
*Sputum culture*	*Streptococcus pneumoniae*, *α*-hemolytic streptococcal species	
